# Society Meetings

**Published:** 1916-01

**Authors:** 


### SOCIETY MEETINGS

**Brief reports and announcements of meetings of societies of Western New York, and those of general scope, are requested from Secretaries. Copy should be on hand the fifteenth of the month. Full transactions will be published at cost of composition.**

**DOCTOR: Is your Society properly represented here? If not, please bring our standing announcement to the attention of the members.**

**The American College of Surgeons** has secured from its Fellows an endowment fund of $500,000, to be held in perpetuity, the income only to be used. The College, which is not a teaching institution but rather a society or a college in the original sense, now lists about 3,400 Fellows in Canada and in the United States. Without precedent for swiftness of development it stands today a powerful factor both in the art and in the economics of surgery.

1. Since the whole problem of the training of specialists for the practice of surgery is the primary purpose of the College, the Regents propose at an early date to present a clear conception of the College to the undergraduate medical students of this continent. The Regents, further, will ask each senior who has in mind to specialize in general or any branch of surgery to register with the College. As these students, then, serve later as internes and as surgical assistants, they will be requested to report these facts. The College, in turn, will systematically seek information as to the ability and character of such men; and the information thus obtained becomes the basis of admission to Fellowship in the College. In addition to this procedure, the Regents will insist upon the proper keeping of case histories, and they will endeavor to stimulate in these men in training right ideals of medical practice. In this program they ask the active co-operation of the faculties of the medical schools and of all practitioners of medicine.

2. Inasmuch as proper training in surgery is inseperably involved with the conduct and efficiency of hospitals, the College will seek accurate data on all matters which relate to hospitals. From time to time it will publish studies upon hospital problems, the purpose being always to be helpful to the hospitals. These publications, further, will inform recent medical graduates as to where they may seek adequate general or special training in surgery. To be concrete the College will deal with such problems as (a) the proper equipment for medical diagnosis, e. g., well equipped laboratories for chemical, pathological, and X-ray work; (b) the proper forms for case histories and the facilities for keeping these records; (c)

the management and the curricula of the nurses training schools; (d) the specialization essential in any well organized hospital.

3. The College will ask the faculties of medical schools to consider the advisability of conferring a supplementary degree of proficiency in general surgery and in the various specialties of surgery.

4. The College will issue readable monographs, educational in nature, to the press, to the general public, to hospital trustees, and to the profession of medicine upon subjects of medical procedure and the whole meaning of fitness to practice surgery.

---

**The Buffalo Academy of Medicine** has held the following meetings since our last report:

Section of Pathology, Nov. 24, Symposium on Diseases of the Kidney: Etiology, John L. Butsch; Histologic Pathology Wm. F. Jacobs; Clinical Pathology, T. B. Carpenter; Symptomatology, De Lancey Rochester; Treatment, Nelson G. Russell. (Note—Several of these papers have been promised for future publication.)

Section of Surgery, Dec. 1, Symposium on Back Ache: Medicine, Allen A. Jones; Surgery, Frank W. McGuire; Orthopaedics, W. W. Plummer; Gynaecology, James E. King; Neurology, Jas. W. Putnam.

Section of Medicine, Dec. 8, Nature and Properties of Various Kinds of Rays, T. F. Cooke, M. E.; Duodenal Diseases, Henry C. Buswell. (Note—These papers have been promised for future publication.)

Section of Obstetrics and Gynaecology, Dec. 15, Abuse of Forceps, P. W. Van Peyma; Indications for the Use of Forceps, N. Kavinoky; Discussion opened by Irving W. Potter and Wm. T. Getman.

---

**The American Society for the Study of Alcohol and Other Narcotics** held its 45th annual meeting in Washington, Dec. 15 and 16. A large number of papers were presented, mostly dealing with the pathology of alcohol, as well as with sociologic and legislative details. Methyl alcohol and tobacco were also included in the program.

---

**The Medical Union** held its regular dinner and meeting Dec. 15, at the Sanatorium of Dr. Geo. H. McMichael of Buffalo, Dr. McMichael being the host. The members were much interested in the equipment of this new location of Dr. McMichael's establishment for the treatment of inebriety and drug addiction. The following officers were elected: Dr. George H. Westinghouse, president; Dr. George H. McMichael, vice president; Dr. A. W. Bayliss, secretary and treasurer. Dr. John C. Thompson read a paper on Mouth Infection and Prophylaxis, which was well discussed.

---

**The Rochester Academy of Medicine** held a section meeting, Dec. 8, at which the following program was presented: Methods of Examination in Gastro-Intestinal Diseases, Wm. V. Ewers; Value of Chemic Analysis of Gastric Contents, Nathan W. Soble.

A regular meeting of the Academy with Section IV was held Dec. 18, at which nominations were made and other business transacted.

---

**The Medical Society of the County of Chemung** held its annual meeting Dec. 21. Beside business which has not been reported, the retiring president, Chas. F. Abbott gave an address and Ross G. Loop presented a paper on Diagnostics of Certain Surgical Lesions of the Kidneys.

---

**The Niagara Falls Academy of Medicine** met Dec. 13, at 9 o'clock in the parlors of the Hotel Imperial. Dr. A. M. Rooker and Dr. Oscar Baer entertained. Dr. Irving W. Potter of Buffalo was the speaker, his subject being Operative Obstetrics with Special Reference to Vaginal and Abdominal Cesarean Section.

---

### Medical Association of Central New York.

Forty-Seventh Annual Meeting of the Medical Association of Central New York, held at the Home of Rochester Medical Association, 33 Chestnut St., Rochester, N. Y., October 21, 1915, 10 o'clock a. m.

Meeting called to order, Dr. Charles O. Boswell, president, in the chair. The president read his annual address to the association.

Dr. Joseph Roby, of Rochester, read a paper (by invitation) entitled: "Therapeutic Uses. Human Blood Serum."

DR. ROBY: I believe in closing that this new product of mercurized horse serum—I have not had any experience with it—but I think it is a dangerous product to use, especially to use intra-venously. I do not see any advantage over bichloride of mercury. I would much sooner put the bichloride of mercury into the patient than horse serum. As to putting the horse serum in the cord, I think there is the same danger,—I mean, mercurized horse serum. Unquestionably, even after the use of spinal serum, there is an inflammation set up in the cord, and I believe mercurized horse serum would be just as apt to set it up, especially as it is used for the especial purpose of setting up an inflammation of the cord, and the obtaining of human serum is a very simple and easy process.

THE PRESIDENT: Gentlemen, this very interesting and suggestive paper of Dr. Roby's is now open to you for discussion, and I hope that all the members here will feel at liberty to join in the discussion as far as they care to. It will, however, be necessary to have a time limit placed upon the amount of discussion, and the Secretary will make a motion to that effect.

SECRETARY BUETTNER: I move you, Mr. President, that the discussion be limited to five minutes for each person.

Motion seconded. Carried.

THE PRESIDENT: I will start the discussion by asking Dr. Hennington, who has had some experience with blood transfusion, to open the discussion.

DR. HENNINGTON: I did not realize that I might be called upon. My name has been associated with the old surgical method of doing transfusion, and I have seen these various new methods, and I would like to go on record as being convinced that probably the citrated blood method will take the place of all these other methods. It is possible that in a very massive transfusion that the old surgical way might still have its place, but in these diseases where you wish to get whole blood frequently, that is, every week or two weeks, or even at longer intervals, that the surgical method is too complicated to apply it. I think that Dr. Roby is to be very much complimented in his introducing this citrated method in Rochester, and I only fear that in a very short time everybody will be doing it.

THE PRESIDENT: Dr. Ross, will you take part?

DR. ROSS: (Toronto) I have been very much interested in this method for the past six or eight months, and I think Dr. Roby's exposition of it has been most complete and most interesting. I quite agree with the Doctor about the citrated method. I think it will take the place of all other methods. We have chills, and they are objectionable, of course, but I do not think they are serious if the proper technique is followed. With respect to this most interesting case of Dr. Roby's, I have one almost identical, although even worse. His blood was as low as fifteen per cent. Blood cultures were negative. The red blood cells were under a million. I feel quite certain it was not primarily pernicious anaemia. I have obtained positive strepto-coccus blood cultures in three cases,—indeed, in all the cases I have tried in the last six months. I agree with Dr. Roby that pernicious anaemia is due to infection, and I think his suggestion that the spleen may be harboring it is correct. Nevertheless, pernicious anaemia is so distinct that sometimes I find it difficult to think the strepto-coccus is responsible. I once more must express my appreciation of Dr. Roby's most excellent paper, and also for your kind opportunity to me to speak.

THE PRESIDENT: Is there any further discussion? Dr. Jones, do you want to say anything on Dr. Roby's paper on using blood serum?

DR. O. E. JONES: I was not fortunate enough to be here, so that I do not know anything about what Dr. Roby's paper was; only, I have seen some of the results of Dr. Roby's transfusions, and the effects have been extremely gratifying. The patients have improved in general appearance and the physical condition is very much benefitted, so that one patient in particular, who was in rather an extreme condition, unable to scarcely be about, could walk with only the assistance of a cane and leaning on some one's arm, and now is able to do the work on his farm.

THE PRESIDENT: Is there any further discussion of Dr. Roby's paper? If not, I will ask Dr. Roby to close the discussion.

DR. ROBY: In reading my paper I did not thank these different men who referred the cases to me, and I desire to do so. This man who was in here a few moments ago was Dr. Jones' patient. Dr. Jones has been rather waiting, but he is getting along all right now. I do not doubt what Dr. Ross says, that when essential pernicious anaemia is established, blood transfusion probably won't do them any good, but one of the points I was trying to make is that we should get to using transfusion in the early cases. Many of these cases are simple anaemia. There must be a beginning of all these cases. There must be a stage of the game when the spleen is quite small, and it must be so with pernicious anaemia. They do not suddenly get a run of temperature of 103 or 104. They must get there gradually. I believe that like all other infectious diseases, if you can get hold of diphtheria early you can cure your case. The same way with tetanus. If that is taken early you can do something. With cerebro-spinal meningitis the serum is of very much more value early in the game. That was one object of my plea that in these cases of simple anaemia, or apparently simple anaemia, when, after giving a trial of iron and arsenic and fresh air and rest they do not improve, that I believe they ought to be transfused. I have not said anything about the method. The method we have been using here is the citrate of soda method. In one of the late journals the citrate of soda got rather a black eye. I don't remember the name of the author of the article. I think all three of his cases acted badly. We have treated twelve or fifteen cases with citrate of soda, and with no bad results at all.

DR. HOWARD L. PRINCE, of Rochester, presented a paper entitled: "Place of Bone Graft in Surgery (with lantern slides).

THE PRESIDENT: The paper of Dr. Prince is now before you for discussion, and I hope any one here will feel at liberty to open the discussion. Dr. Howk, will you discuss the paper?

DR. HOWK, (Rochester): I have seen some of the results of Dr. Prince's work, and they have all been highly satisfactory. I think that his ability to deal with these cases leads many of us to refer our cases directly to him. In fractures and in diseased conditions of the bone his technique is so far superior to anything that we can indulge in that it seems to me well to give him the opportunity of taking care of them.

DR. SEELYE W. LITTLE, (Rochester), read a paper entitled: "Ductless Glands of a Typical Growth."

THE PRESIDENT: Gentlemen, this discussion on this most suggestive and interesting paper of Dr. Little's will be postponed for a few minutes until Dr. Ross is able to demonstrate to you the use of the diarsenal. The preparation has been made and should not oxydize too long, and the patient is getting nervous.

Dr. Ross demonstrated the use of the preparation referred to.

The President called upon Dr. Stockton, of Buffalo, to open the discussion on Dr. Little's paper.

DR. STOCKTON: Mr. President and Gentlemen: I feel that this is an honor which I am unable to fully justify, to reply to the very thoughtful paper of Dr. Little. There is so much in this paper that challenges one's thought that I feel that no one could attempt a full discussion of it without study of the paper. I do not know what to say to Dr. Little's theory. I think, however, that he saves himself, because he does not stop with theories, he says that it works. Now, if the pragmatic side is sufficient, we are to be congratulated in having this new thing brought before us, regardless of the reasons therefor. It seemed to me that all of the various discussions as to the nature, the possible nature, of the cause of cancer have been faulty, in the fact that they have not been able to show results, and for my own part, most of the theories that have to do with the behaviour of embryonic cells in this extraordinary manner are connected with one well known fact in biology, and that is, it would seem that this disturbance of embryonic cells, to make this extraordinary growth, must be a very widespread influence, since we find cancer in all mammals and all vertebrates, and it is not, perhaps, going too far to find that similar growths occur in the vegetable world. When one realizes this fact—and there are many who believe that that statement is a fact—it seems strange that we have to explain the development of these growths and this stimulation of the embryonic cell through the action of some kind of internal secretion. I do not want Dr. Little to understand that I am trying to combat his statement. I am not. On the contrary, I am very much interested, but I am trying to have him in his reply satisfy some of the objections that arise in my mind, to not only his theory, but all the theories that I have especially heard brought forward for the origin of cancer except by the action of some external agent. I cannot understand, sir, how it is that there can be any common defect in our own natural nutrition which would extend through such wide ranges of animal life, through so many families, which should not have been long ago weeded out, long ago extinguished through the action of natural selection. If there is a cause that produces a definite effect like cancer, and a thing which extends through so many families, it must be a thing of the earliest possible origin, it must go back to the very beginning of animal life. Now, it seems to me that a thing so definitely detrimental to the development of animal life, if it depends upon one of those defects, would have been excluded by Darwin's law long ago. I am very much interested in seeing the Doctor's actual demonstration, and from which he implies that he has seen good results from this treatment. I think that is worth more than we can all say, and if this study of the internal secretions has led him to a view which in part will produce good results, I think that is admirable and he is to be most heartily congratulated.

DR. BENEDICT (Buffalo): Mr. President, there is just one thing I would like to call attention to in this matter, and that is, I think we can say that the one fact definitely shown with regard to the etiology of cancer is that it occurs most frequently with advancing years. As I remember it, the maximum is about eighteen per cent for women of about the age of fifty-five to sixty, that is, eighteen per cent of all deaths at that age; about ten per cent less for men. There is really no sexual difference, if we allow for the fact that the female has two vulnerable organs, the uterus and the breast, which are not present in a male. Now, in spite of this fact that age seems to be the point in the etiology of cancer that we can all agree upon, and that is established by statistics, there are certain curious fallacies. One is, that the incidence does not increase as age increases, that is, as we pass the point, of about sixty for women and about fifty-five for men, the incidence goes down, and in very advanced ages we find that the relative number of deaths from cancer is only about that which we find at twenty-five to thirty years of age. Here we have an apparent cause, but increasing the cause does not increase but rather decreases its operation. Now, in another respect let us analyze; take the different things which we say make up old age, or, regard the matter in the very superficial way, do our cancer patients look old? Are they old for their years? Now, in my observation, as a rule they are not. I would not say that cancer occurred in people who apparently had not reached their actual age, and, on the other hand, I think we can say that senility relative to actual age is not a particular cause of cancer. Neither do we find cancer in patients in which we can pick out any one factor of old age, as, for instance, a tremor, or a condition of the arteries, or grey hairs, or any other one indication of age. Now, there is a curious paradox that occurs to me, that Dr. Little may have put us on the right track by saying that age is a factor in the etiology of cancer only because it gives chances for something else to develop. The old saying is that the pitcher that goes too often to the well is broken, and the person who keeps on living will develop these causes if he has time. I think we ought to bear these facts in mind and check them off in our experience. I for one am deeply indebted to the essayist for this point he has given.

DR. LITTLE: I had rather hoped there would be bitter criticism. I did not read the table at the time. Some of these points have been met at that. For instance, cancer occurs practically universally in the animal kingdom,—maybe in the vegetable kingdom; that is doubted; I would say also that the ductless glands occur universally in every creature, so far as is known, that has cancer. Ductless glands are a very early development biologically, and they increase with the specialized tissues.—The defect should have been eliminated by natural selection: It would have been, according to the theory, eliminated by natural selection, except for the fact that civilization—By the way, cancer increases as the complexity of civilization increases—it would have been eliminated by natural selection, but we are deliberately engaged in our business as physicians in trying to go counter to natural selection, and we keep alive all the defectives we can, in every way. Further, the more highly specialized we become the more depends, according to the theory, on the ductless glands, which have a specializing function. Therefore, it will not be eliminated by natural selection, granting that the theory be true, until they get specialized so far that many of us fail, and more and more are failing all the time in this particular respect. I thought the last speaker was going to jump on me in regard to the odor we got. Both those points are mentioned in this table of cancer on one side and ductless glands on the other. For instance, the age incidents of cancer are well known, middle age especially, and after. As a person gets older ductless glands, or anything else, fail. If ever, that is the time they are going to fail. It is rare in the young, but it is very rapid when it does occur in the young, naturally. In the young of the ductless glands it must be more serious than in the old, because metabolism and growth are most active and the ductless glands are more active. Diabetes is another illustration. Diabetes in the young is, in the result, much more serious by far than diabetes in the old. We have old looking patients. A cancer patient—I think that is perfectly true—take them just as they come, they do not look any older than their years. That is nothing. That does not prove that their ductless glands are older than they ought to be. All this whole subject of ductless glands is recent, and there is one very great proof that I lack and I cannot get, and there is no reference to it in the literature, and that is autopsies. I would like to get an autopsy on every patient that dies of cancer in Rochester, with reference to the ductless glands. If I could get that, and if it showed right straight along, it would be fairly valuable. I have only a few and those are positive. I have a patient with cancer of the skin, perfectly operable. He declines and says he will die with it. He came to me because he knew I had a cure for cancer. I told him he was mistaken, that I would try him for a little while, and if the thing got worse I would decline to have anything more to do with him. He got better and it disappeared, and we stopped the medicine. He came back in about three or four months with the cancer back again. We started again and it went away. That is all I can say. The Lord knows what is going to happen to that man. I don't yet. The Doctor says I say it works. Sometimes. Most times not, but there is enough for me—I am one of these faddists—there is enough for me to be positive that there is some relation between those things. I have seen it too many times.

(To be continued in next issue).

---

### Medical Society of the County of Erie.

The 94th Annual Meeting of the Medical Society of the County of Erie was held on December 20th, 1915, at Townsend Hall, 25 Niagara Square, Buffalo, N. Y.

President Hurd called the Society to order at 6:30 p. m.

President Hurd announced that the annual election of officers would be held at this meeting and called for any further nominations to those which had already been made at the regular October meeting. On motion of Doctor Hopkins nominations were declared closed, no further nominations being made. The motion was carried.

President Hurd then appointed the following tellers: Doctors Woodruff, Sharp and Lytle.

Treasurer Lytle submitted his annual report, which showed the number of members in good standing as 647. Number of members who died during the year 4. Number resigned 0. Removed to other counties 4. Retired 4. Number of members in arrears with their dues 79. Total receipts during the year was $3426.07. Of which there was a balance on hand of $1514.00, the remainder having been paid in dues to the Medical Society of the State of New York and for current expenses. The Treasurer's report was adopted.

The Secretary then read the minutes of the regular meeting held October 18th, 1915, and also the minutes of the Council held December 18th, 1915, both of which were adopted as read.

Doctor William F. Jacobs, Chairman of the Committee on Membership reported favorably upon the following list of applicants, each of whom were separately ballotted for and elected: Dr. Albert A. Gartner, 715 Broadway; Dr. Carlton E. Wertz, 1440 Jefferson street; Dr. Frank Kruse, Moses Taylor Hospital; Dr. Herbert E. Wells, Lackawanna, N. Y.; Dr. Herbert H. Bauckus, 113 High street; Dr. August Lascola, 539 Michigan avenue; Dr. Baldwin Mann, 815 Auburn avenue; Dr. John Ludwig, 522 Best street; Dr. Arnold H. May, 177 Walnut street; Dr. Harvey P. Hoffman, 1451 Jefferson street.

President Hurd expressed the thanks of the Society to Chairman Jacobs for his activity in securing new members during the past year.

Reports of officers were then called for.

Doctor John D. Bonnar, Chairman of the Board of Censors presented the report of the Board and also that of the Attorney, Mr. Alfred L. Harrison. An interesting feature of Chairman Bonnar's report was that among the persons prosecuted for the illegal practice of medicine was a Chiropractor, who was fined $25.00, which the Censors considered as establishing a precedent or record, this being the first conviction. Doctor Bonnar was given a vote of thanks for his activity during the past year.

Doctor Henry R. Hopkins, Chairman, Committee on Public Health, submitted his annual report, which was a sort of review of the activities of this Society in the matter of securing better medical education and a higher medical standing. In 1871 the Medical Society of the County of Erie began the first agitation, which finally resulted in passing a State law in 1891 requiring a State license for the practice of Medicine. Doctor Hopkins' report then went into economic, hygiene, and other conditions, and ended by submitting the following resolution:

RESOLVED, that the Medical Society of the County of Erie, State of New York, hereby makes record of its conviction that to Americans the subject of Forestry is a matter of enormous vital significance; that to the medical profession of America the subject should appeal as to no other group of our citizens; that record should be made and repeated by medical societies, county, state and national, urging upon those in authority the importance of the most intelligent study of this question of Forestry to the end that suitable action by our governments, state and national, be taken in this our most vital problem.

RESOLVED, that these resolutions be transmitted under the seal of this Society to the Medical Society of the State of New York, with the request that the same receive due consideration and if approved that the matter be brought to the attention of the American Medical Association at its next meeting.

Resolutions as submitted were adopted and the entire report was then approved.

President Hurd expressed the appreciation of the Society to Doctor Hopkins for his valuable report.

Doctor John B. Woodruff, Chairman of the Committee on Economics then submitted his annual report. This was the first report since this committee was created. It was a very exhaustive resume of contract practice in this City of which more than 230,000 people are obtaining medical service at a nominal cost of $1.00 per year. The report was adopted with the thanks of the Society.

On motion of Doctor Bonnar the polls were declared closed and the tellers then presented the result of the ballot, by which the following were declared duly elected:

President—Doctor Franklin W. Barrows.

First Vice-President—Doctor Irving W. Potter.

Second Vice-President—Doctor John C. Thompson.

Secretary—Doctor Franklin C. Gram.

Treasurer—Doctor Albert T. Lytle.

Censors—Doctor John D. Bonnar, Doctor Francis E. Fronczak, Doctor Arthur G. Bennett, Doctor Archibald D. Carpenter and Doctor Frank A. Valente.

Chairman, Committee on Legislation—Doctor Harvey R. Gaylord.

Chairman, Committee on Public Health—Doctor Nelson G. Russell.

Chairman, Committee on Membership—Doctor William F. Jacobs.

Chairman, Committee on Economics—Doctor John V. Woodruff.

Delegates to the State Society—Dr. Arthur G. Bennett, Dr. Albert T. Lytle, Dr. Julius Richter, Dr. Franklin W. Barrows.

The President then called President-elect Barrows to the chair and read his annual address.

The Presidential address was a resume of some of the most important progress in scientific medicine.

The thanks of the Society was tendered the retiring President after which the meeting adjourned.

